# Microwave-Assisted Synthesis, Characterization and Modeling of CPO-27-Mg Metal-Organic Framework for Drug Delivery

**DOI:** 10.3390/molecules26020426

**Published:** 2021-01-15

**Authors:** Anton I. Kudelin, Konstantinos Papathanasiou, Vera Isaeva, Juergen Caro, Tapio Salmi, Leonid M. Kustov

**Affiliations:** 1 Russian Academy of Sciences, N. D. Zelinsky Institute of Organic Chemistry, Russian Federation, Leninsky Prosp. 47, 119991 Moscow, Russia; kudelin.anton@gmail.com (A.I.K.); sharf@ioc.ac.ru (V.I.); 2 International Laboratory of Nanochemistry and Ecology, National University of Science and Technology MISiS, Moscow, Leninsky Prosp. 4, 119991 Moscow, Russia; k.papathanasiou@uoc.gr; 3 Laboratory of Nano and Quantum Engineering, Leibniz University Hannover, 30167 Hanover, Germany; juergen.caro@pci.uni-hannover.de; 4 Faculty of Science and Engineering, Abo Akademy University, FI-20500 Turku, Finland; tsalmi@abo.fi; 5 Faculty of Chemistry, Lomonosov Moscow State University, 119992 Moscow, Russia

**Keywords:** metal-organic frameworks, CPO-27, drug delivery, paracetamol, aspirin, microwave synthesis

## Abstract

The coordination polymer CPO-27-Mg was rapidly synthesized under microwave irradiation. This material exhibits a sufficiently high drug loading towards aspirin (~8% wt.) and paracetamol (~14% wt.). The binding of these two molecules with the inner surface of the metal-organic framework was studied employing the Gaussian and Plane Wave approach of the Density Functional Theory. The structure of CPO-27-Mg persists after the adsorption of aspirin or paracetamol and their desorption energies, being quite high, decrease under solvent conditions.

## 1. Introduction

In the modern world, the development of medicines is becoming increasingly difficult because of the rising cost of research work, production costs, necessity to reach a high extent of purification, licensing, and associated expenses. Therefore, scientists pay attention to new ways to apply already invented and well-known drugs, and one of such ways is drug delivery with controlled or sustained release of the biologically active substance. There is a number of vehicles for this purpose: various polymers [1], liposomes [2], and nanoparticles [3] are among them. Porous hybrid solids—metal–organic frameworks (MOFs) have been studied in view of potential application for drug delivery for almost ten years [4], and some outstanding results have been achieved. For instance, MIL-88A and MIL-100 effectively entrap busulfan and show no cytotoxicity [5] due to their bio-friendly building blocks: MIL-88A consists of iron(III) and fumaric acid, and MIL-100 consists of iron(III) and trimesic acid, which is believed to be one of the least harmful MOF linkers as other benzenecarboxylic acids [6]. A metal–organic framework should meet several criteria to be suitable for the role of a drug carrier: (a) it should contain no hazardous components, especially heavy metals, (b) it must have a sufficient pore limiting diameter (PLD) to be able to encapsulate a drug from solution, and (c) the drug loading on its surface should be significant. Also it is desirable that (d) a drug could be able to leave the pores without the decomposition of the crystal and (e) MOF can form nanocrystals to ease intravenous administration. CPO-27-Mg (CPO stands for Coordination Polymer of Oslo) [7] consists of magnesium cations and residues of 2,5-dihydroxyterephthalic acid (DOBDC) and forms a framework with one-dimensional pores (PLD 10.76 Å), and is known to form nanocrystals under mild conditions [8], thus it fits into the (a), (b), and (e) criteria. Although this MOF has been already noted as a potential drug carrier [6], it has not been studied in this application yet unlike CPO-27-Ni [9] and cationic CPO-27-Fe [10]. There is only one paper devoted to the use of the CPO-27(Mg) coordination polymer in drug delivery of hydrogen sulfide [11]. No publications can be found on the adsorption of aspirin and paracetamol on CPO-27 materials. The purpose of the current study is to show that this MOF is unfairly ignored because it fits into the (c) and (d) criteria of the ideal drug carrier as well. We employed powerful and popular modern tools of the solid state density functional theory (DFT) to explore energy effects of binding two trial well-known drugs—2-acetoxybenzoic acid (aspirin) and *N*-acetyl-para-aminophenol (paracetamol) inside the pores of CPO-27-Mg.

## 2. Results and Discussion

The logics of the paper were as follows:

(1) we chose CPO-27(Mg) as the most suitable MOF structure from the point of view of its potential toxicity (it is the least toxic among similar structures) and pore volume/pore size characteristics that need to be suitable to accommodate as much drug as possible,
(2) then we performed DFT calculations to predict the behavior of the two drugs in the chosen CPO-27(Mg) structure,
(3) then we synthesized the selected CPO-27(Mg) structure and chose the microwave (MW) method, based on our previous experience on the microwave synthesis of MIL-100, MIL-53, and ZIF-8 structures and some literature data [12,13,14] that demonstrates that the MW synthesis provides certain advantages over the conventional solvothermal protocol, such as reduced duration of synthesis, reduced temperature of the synthesis, reduced pressure (instead of the autogenous pressure we used 1 atm), and controllable and uniform particle size,
(4) and finally, we performed experiments on adsorption of aspirin and paracetamol in the predicted and synthesized CPO-27(Mg) structure.

### 2.1. DFT Simulations

Convergence tests were performed to evaluate the reliability of DFT functionals in the case of CPO-27-Mg. Commonly used in a periodic case, the functionals HSE06, PBE0-D3, PBEsol with and without Grimme’s correction were chosen for tests. ωB97X-D and vdW-DF2 were studied in this benchmark as well in order to find out how the separation of range and the introduction of non-local dispersion forces affect the model of the MOF cell. In addition, in the original study vdW-DF2 was shown to return right adsorption energies for a MOF of the CPO-27 type. Four of six studied functionals employ that type of force as it is well known that dispersion strongly influences the processes in a MOF cell and its geometry. They are summarized in Table 1. Relative errors in the calculation of crystallographic parameters were chosen as criteria.

All functionals tested overestimate the *a*, *b*, and *c* parameters, while β and γ are almost exact: the error being within one per cent. Surprisingly, hybrid HSE06, PBE0 with Grimme’s correction, and range-separated ωB97X-D are unreliable in that case and give significantly larger cells compared to the experiment. The results obtained with non-local vdW-DF2 are noticeably better, and the cell volume differs only by 4%. The best performant is PBEsol, and Grimme’s correction justifies its introduction improving the result. Due to the benchmark we chose PBEsol-D3 as a tool for further study of CPO-27-Mg. The knowledge of the energy of drug desorption from the MOF porous structure (E_des_) is important for understanding the kinetics of this process. At energies E_des_ >> 0, the explored system is chemically stable and consequently a drug will be released only with MOF decomposition. At energies E_des_ ≤ 0, the desorption is diffusively controlled and more pharmacologically useful in contrast to the previous case due to the ability of small molecules to leave pores without a breakdown of the adsorbent. The energy of desorption without thermal effects can be represented as:

E_des_(g) = E_tot_(MOF) + E_tot_(*m*) − E_tot_(*m*@MOF) (1)

where E_tot_ denotes the total electron energy of a molecule (*m*), MOF, and the host-guest complex (*m*@MOF). Although Equation (1) is valid for desorption to the gas phase, it does not take into account solvent effects, which are present in the organism environment and moreover can be the driving force of the desorption. The Equation (1) was modified into:

E_des_(s) = E_tot_(s@MOF) + E_tot_(sm) − E_tot_(sm@MOF) − [N(s@MOF) + N(sm) − N(sm@MOF)]E_tot_(s) (2)

Dealing with the total energies of MOF with pores filled with a solvent (s@MOF), a solvated molecule (sm), the host-guest complex with the solvent in the pores (sm@MOF), and the solvent molecule (s); N is the number of solvent molecules in s@MOF, sm, and in sm@MOF, respectively. Although the choice of N might be based on crystallographic data, it is missed for the case of sm@MOF. Besides, it is quite difficult to find the global minimum of such a weakly coupled system, and even the evaluation of the average energy requires a molecular dynamics run over a long period of time that has an extreme computational cost. Therefore the SCCS method which has shown to be reliable in similar cases was employed. To achieve better accuracy of this approximation, solvent molecules with known displacements were added to the model into the coordination sphere of unsaturated metallic sites. For the 2 × 1 × 1 supercell of CPO-27-Mg, N(s@MOF) was assigned as 12 by the number of Mg atoms, N(sm) was 0, thus the solvent was replaced by the SCCS model, N(sm@MOF) was equal to 10 for the complex of CPO-27-Mg with aspirin (Asp@CPO-27-Mg), and to 11 for the complex with paracetamol (Par@CPO-27-Mg).

**Figure 1:**
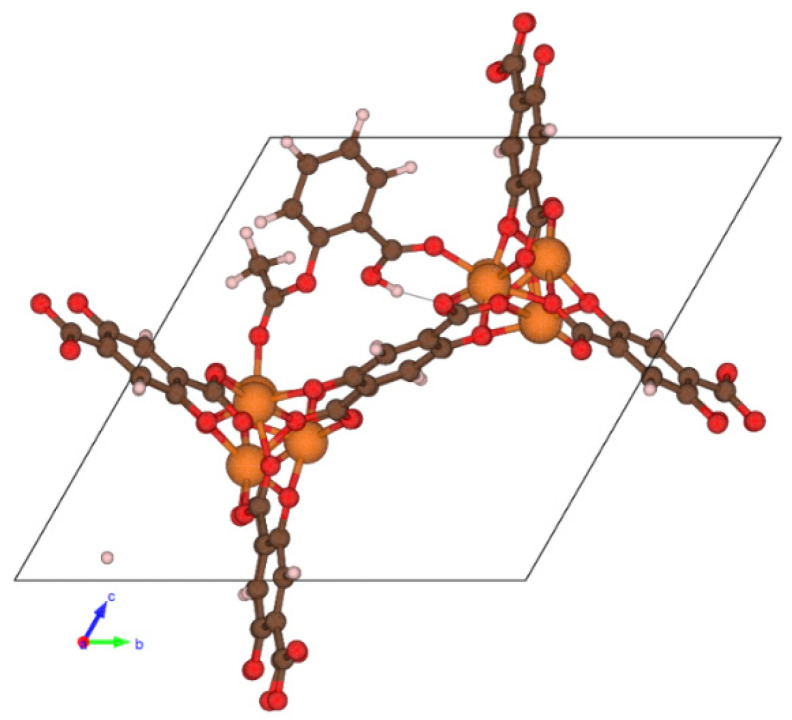
Projection of the CPO-27-Mg cell with aspirin, no solvent is shown.

The geometries of complexes of two considered drugs—aspirin and paracetamol—with CPO-27-Mg are rather predictable: adsorption has a coordination nature and is carried out via bonding a molecule to MOF metallic sites. Two carbonyl groups of aspirin form two coordination bonds C=O—Mg, and the hydroxyl group of the carboxy group forms a hydrogen bond with oxygen of the Mg—O site (Figure 1).

**Figure 2:**
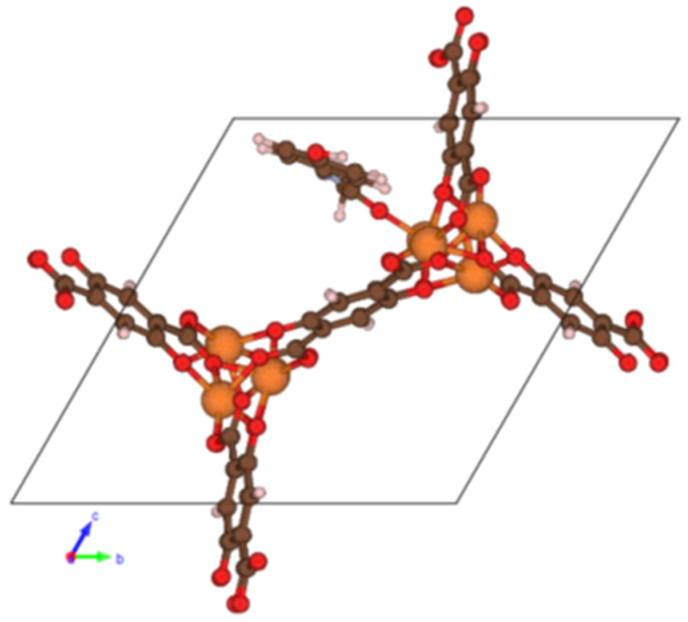
Projection of the CPO-27-Mg cell with paracetamol, no solvent is shown.

The only coordination bond of paracetamol with the surface of CPO-27-Mg occurs due to the presence of the carbonyl group in the molecule (Figure 2); the hydroxyl group is bound by water molecules. Based on general ideas, one can guess that the desorption of the triple bound aspirin should be more complicated and therefore less energetically beneficial than the desorption of single bound paracetamol.

However, in our case of desorption to water, computational results (Table 2) gave −5.6 kJ mol^−1^ of E_des_ (water) for aspirin and 51.8 kJ mol^−1^ for paracetamol. The discrepancy of the guess and our results can be explained by strong solvation effects which were evaluated as E_des_ (water) − E_des_(g) giving −238.1 kJ mol^−1^ for aspirin and −118.7 kJ mol^−1^ for paracetamol. Although the contribution of dispersion forces to E_des_ (water) is significant, 42.4 and 60.0 kJ mol^−1^ for aspirin and paracetamol, respectively, most of the binding energy comes from covalent interactions. These values are in agreement with those reported previously by Horcajada et al. for the case of drug-containing complexes of MIL-53(Fe) where only dispersion interactions occurred [4,5,6]. Thus, the desorption of these molecules to a gas phase is almost impossible, they can be released from the MOF pores to water, wherein Asp@CPO-27-Mg flawlessly suits the criterion on E_des_, and despite the higher value of E_des_(water) Par@CPO-27-Mg still can be used as a drug delivery complex.

### 2.2. Synthesis and Adsorption

**Figure 3:**
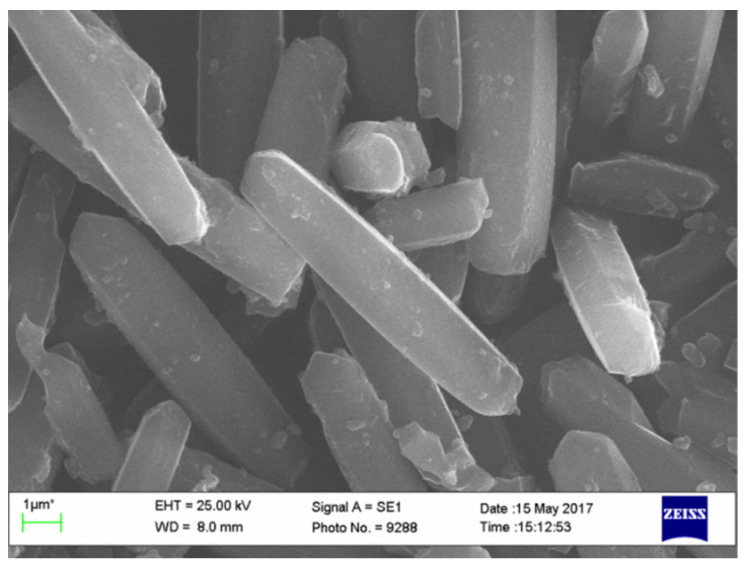
SEM image of crystallized CPO-27-Mg.

To ensure that drugs actually enter the MOF pores, we conducted several synthetic experiments. First, we obtained DOBDC by a cheap and simple procedure using hydroquinone, CO_2_, formic acid, and K_2_CO_3_ as starting materials. Then microwave irradiation was employed to carry out the rapid synthesis of high-quality CPO-27-Mg, which had a specific surface area of 1007 m^2^/g after the microwave activation procedure. Crystallites of the obtained material were of a stretched shape and the size was about 8.8 × 1.8 µm^2^ (Figure 3). Although these sizes do not allow the use of the material for intravenous administration, it is still worthwhile to try them for the adsorption study.

**Figure 4:**
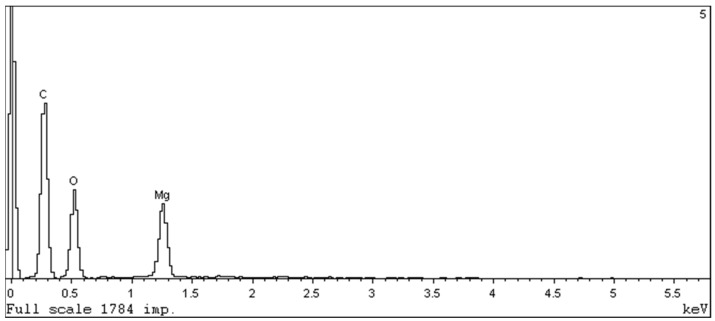
EDX spectrum of crystallized CPO-27-Mg.

**Figure 5:**
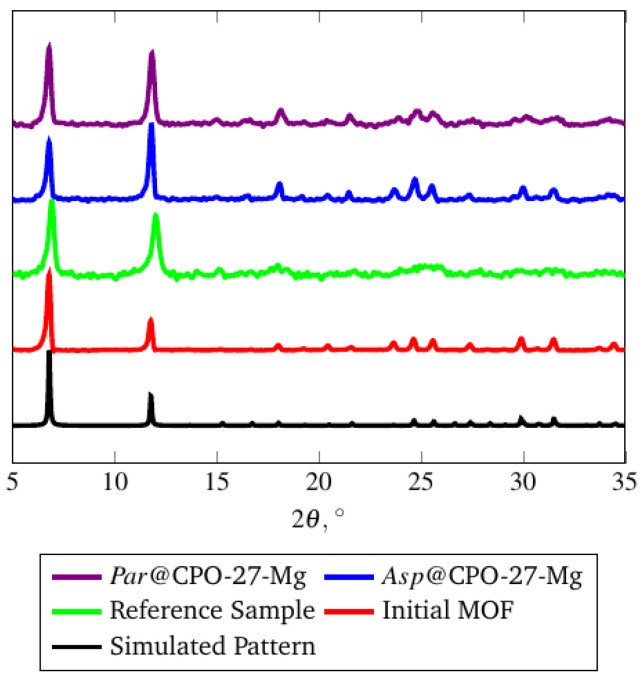
Powder X-Ray Diffractograms of CPO-27-Mg samples. Reference sample was CPO-27(Mg) prepared by the solvothermal method. Initial MOF was CPO-27(Mg) prepared by the microwave method.

The EDX spectrum (Figure 4) shows that no nitrogen was left in MOF and all DMF was successfully removed during the activation procedure. PXRD of the obtained material is in good agreement with the simulated pattern (Figure 5).

The study of the CPO-27(Mg) sample by diffuse reflectance Fourier-transform IR spectroscopy confirms the presence of 2,5-dihydroxy-1,4-benzenedicarboxylate linkers in the structure. The broad bands in the region of 3500–3300 cm^−1^ are assigned to hydrogen-bonded phenolic hydroxyl groups. The intense absorption bands in the regions of 1628 and 1439 cm^−1^ belong to the antisymmetric and symmetric stretching vibrations of the carboxylate groups. Absorption bands in the region of 1516 and 1593 cm^−1^ correspond to aromatic ring vibrations, and the bands of weak intensity in the region of 3067 cm^−1^ belong to the stretching C-H vibrations of the aromatic system.

Using CPO-27-Mg as a host framework, complexes of aspirin (Asp@CPO-27-Mg) and paracetamol (Par@CPO-27-Mg) were prepared. Drug loadings were examined employing elemental analysis with a reference sample obtained at the same conditions and were found to be equal to 8 wt.% of the MOF weight for aspirin and 14 wt.% for paracetamol. Although the results are far from those observed in the case of mesoporous MOFs such as MIL-100-Fe, they are quite good and exceed the loadings in commonly used non-MOF drug carriers. The drug amounts in the pores correspond to one aspirin molecule per one 3 × 1 × 1 supercell of CPO-27-Mg, and one paracetamol molecule per one 1.5 × 1 × 1 supercell and they are conformed to E_des_(ethanol) obtained in DFT simulations (Table 2). Consequently, ab initio calculations can be used for evaluation of drug loadings, although calibration for energies may be required. As mentioned previously, structures of the CPO-27 type are rigid and the effect of “breathing” was not observed when small molecules were adsorbed at their surface, and the main reflections of powder patterns of the obtained host-guest complexes are the same as in the initial MOF (Figure 5). This fact is in agreement with computational results showing no cell volume changes in both cases (Asp@CPO-27-Mg and Par@CPO-27-Mg). It is noteworthy that the BET specific surface area of the samples containing 8% of aspirin and 14 wt.% for paracetamol reduced from 1007 m^2^/g for the initial CPO-27(Mg) sample to 625 m^2^/g and 505 m^2^/g, respectively.

**Figure 6:**
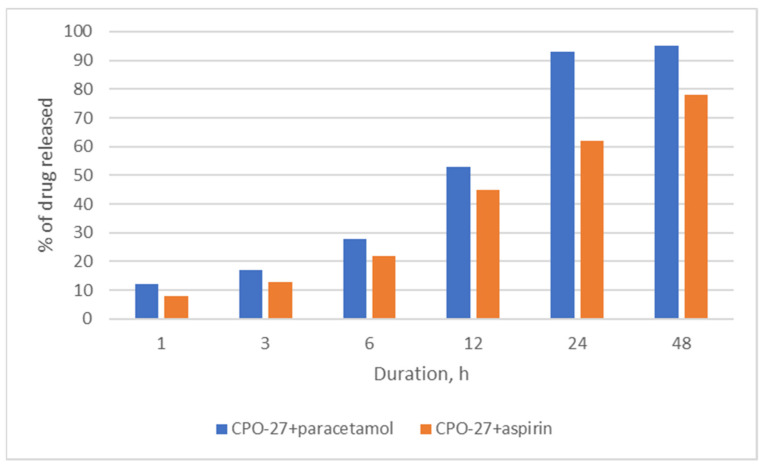
Release of aspirin and paracetamol from the pores of the CPO-27 material.

The data on the release of the encapsulated drug molecules from the pores of the CPO-27(Mg) material are presented in Figure 6. It is seen that the release of paracetamol occurs at a higher rate and reaches about 95% of the amount encapsulated by the MOF, whereas the release of aspirin takes longer times (over 48 h) and does not approach the nearly complete release of the drug. This is consistent with the DFT calculation results showing that aspirin is adsorbed stronger in the CPO-27(Mg) materials than paracetamol, even though the amount of adsorbed aspirin is somewhat lower compared to paracetamol.

## 3. Materials and Methods

### 3.1. Computational Details

All quantum chemistry calculations were carried out employing Goedeker–Teter–Hutter dual-space pseudopotentials [15] and the double-ζ valence polarized correlation-consistent basis sets using the Gaussian and Plane Wave approach implemented in the CP2K 4.1 package [16]. The kinetic energy cut-off for plane wave basis set were set to 800 Ry, sampling of Brillouin zone was limited only to Γ-point due to the fact that the unit cell is large enough to provide required precision. The geometry optimization of all systems was performed with the use of the Broyden–Fletcher–Goldfarb–Shanno algorithm and its limited memory version [17] when the self-consistent continuum solvation (SCCS) model [18] was applied. Broyden and Pulay mixing procedures were used as density updating methods. Cell and ionic relaxations were allowed without any geometry restrictions, stress tensors were calculated analytically, and default convergence criteria were chosen. SCCS calculations were conducted with fixed cell vectors due to limits in the method implementation. The initial structure of CPO-27-Mg was derived from the Computation-Ready, Experimental (CoRE) MOF Database [19] (VOGTIV_clean_h entry), guest molecules were added using the Avogadro program [20]. Convergence tests were performed using HSE06 [21], PBEsol [22], PBE0 [23], ωB97X-D [24] exchange-correlation functionals from LIBXC [25], vdW-DF2 [26] was constructed by the use of rPW86 exchange from the same library. The DFT-D3 dispersion correction was used explicitly for PBE0 and PBEsol [27]. The entry VOGTIV_clean_h of CoRE-MOF was taken as the initial guess without any changes.

### 3.2. Material Synthesis

2-Acetoxybenzoic acid (aspirin) and *N*-acetyl-p-aminophenol (paracetamol) were purchased from Acros Organics. 2,5-Dihydroxyterephthalic acid (DOBDC) was synthesized from hydroquinone by a procedure suggested by Cadot et al. [28]. Dimethylformamide (DMF) was distilled over calcium hydride before using. All other reagents were purchased from Acros Organics and used without any further purification. To synthesize CPO-27-Mg, solutions of 2.54 mL of Et_3_N and 0.9 g of 2,5-dihydroxyterephthalic acid in 50 mL of water, 2.33 g of magnesium nitrate hexahydrate in 50 mL DMF were quickly mixed in a 250 mL beaker, which was then subjected to microwave irradiation for 10 min in the microwave oven Landgraf MW4000 at 2.45 GHz and 10% of the full power (80 W). The precipitate was centrifuged, washed three times with water and ethanol, and dried at 70 °C overnight yielding 0.917 g (yield 90%) of a yellow powder. The guest molecules incorporated in the crystals were removed under a dynamic vacuum at 150 °C for 12 h. Host-guest complexes were prepared by the following general procedure: 4 mL of saturated ethanol solution of a drug were vigorously stirred with 0.1 g of MOF for 24 h under ambient conditions. The obtained materials were centrifuged and dried at 70 °C overnight. Pure ethanol was used for control sample preparation.

In order to monitor the release of the drug from the pores of the CPO-27 material, the sample with pre-adsorbed aspirin or paracetamol (100 mg) was placed in 100 mL of deionized water at 37 °C under continuous stirring (50 rpm). At different incubation times, 20 mL of supernatant was recovered by centrifugation and replaced with the same volume of fresh deionized water at 37 °C. The amount of released drug was determined by MS-GC using a FOCUS DSQ II chromato-mass-spectrometer with a TR-5ms capillary column.

### 3.3. Material Characterization

To verify the purity of extracted drugs, a nuclear magnetic resonance spectrometer Bruker Avance 400 and the GSim program as a spectral analyzer were used. To characterize and analyze the initial sample of CPO-27-Mg, X-ray diffraction (PXRD), scanning electron microscopy (SEM), and energy-dispersive X-ray spectroscopy (EDX) methods were employed. Adsorption measurements were conducted by the Klyachko-Gurvich method [29]. Other synthesized materials were tested with the use of elemental analysis and PXRD methods. Elemental analysis of the as-synthesized CPO-27(Mg) prepared under microwave activation after overnight drying at 70 °C gave the formula C_8_H_4_O_4_Mg corresponding to the ideal composition of this material. PXRD experiments were carried out at room temperature using a DRON-2 diffractometer with Cu Kα (λ = 1.5406 Å) radiation and a step of 0.02° in 2θ for the angle interval 5–35° with Fityk [30] as a processing program. SEM images and the EDX spectrum of the graphite coated sample of CPO-27-Mg were obtained using a Supra 50 VP LEO instrument with the INCA Energy+ Oxford microanalysis system. Spectra of aspirin and paracetamol are respectively: δH (400 MHz; DMSO-*d*_6_; Me_4_Si) 3.02 (3 H, s, Me), 7.32 (1 H, d, 5-H, *J*_5,4_ = 7.8), 7.41 (1 H, t, 3-H, *J*_3,4_ = 7.4), 7.67 (1 H, ddd, 4-H, *J*_4,5_ = 7.8, *J*_4,3_ = 7.4, *J*_4,6_ = 1.6), 7.98 (1 H, dd, 6-H, *J*_6,5_ = 7.8, *J*_6,4_ = 1.6), 13.16 (1 H, s, OH); δH (400 MHz; DMSO-*d*_6_; Me_4_Si) 2.00 (3 H, s, Me), 6.70 (2 H, d, CH, *J* = 9.0), 7.36 (2 H, d, CH, *J* = 9.0), 9.16 (1 H, s, OH), 9.67 (1 H, s, NH). Spectra of 2,5-dihydroxyterephthalic acid are: δH (400 MHz; DMSO- *d*_6_; Me_4_Si) 7.62 (s); δC (100 MHz; DMSO-*d*_6_; Me_4_Si) 117.56, 119.55, 152.22, 170.57.

## 4. Conclusions

Thus, our work shows that CPO-27-Mg is an excellent choice for the preparation of new MOF-based drugs for biomedicine. It contains no harmful components and has a sufficiently high drug loading capacity towards aspirin and paracetamol, which should be able to leave the pores in the presence of water. Microcrystalline CPO-27-Mg can be rapidly prepared by microwave-assisted synthesis from cheap compounds with a good yield reaching at least 90% within 10 min of the MW synthesis at 1 atm, compared with the duration of 30–70 h, elevated (autogenous) pressures of 15–30 atm from the same precursors.

## Figures and Tables

**Table 1:** Relative errors in determining the crystallographic parameters of the CPO-27-Mg cell using various density functional theory (DFT) functionals.

Functional	Δ*a/a*_0_, %	Δ*b/b*_0_, %	Δ*c/c*_0_, %	Δ*α/α*_0_, %	Δ*β/β*_0_, %	Δ*γ/γ*_0_, %	Δ*V/V*_0_, %
HSE06	−11.62	−6.25	−4.44	−1.64	0.93	−0.35	−24.43
PBEsol	−1.25	0.09	−0.01	0.04	0.07	−0.15	1.02
revPBE	−2.77	−0.87	−0.87	−0.17	0.16	−0.18	−4.51
PBEsol-D3	−0.77	−0.16	−0.06	−0.04	0.00	−0.14	−0.45
revPBE-D3	−2.35	−0.74	−0.80	−0.01	0.08	−0.17	−3.80

**Table 2:** Energies of molecules desorption to a solvent, kJ/mol.

Complex	E_des_ (Water)	E_des_ (Ethanol)
Asp@CPO-27-Mg	−5.6	0.7
Par@CPO-27-Mg	51.8	77.9

## Data Availability

Data are available from the correspondence authors.

## Sample Availability

Samples of the compounds are available from the authors.
